# Sleep in Wilson’s disease: Questionnaire based study

**DOI:** 10.4103/0972-2327.78047

**Published:** 2011

**Authors:** Archana B. Netto, Sanjib Sinha, Arun B. Taly, Samhita Panda, Shivaji Rao

**Affiliations:** Department of Neurology, National Institute of Mental Health and Neuro Sciences, Bangalore, India; 1 Department of Biostatistics, National Institute of Mental Health and Neuro Sciences, Bangalore, India

**Keywords:** Epworth sleepiness scale, Pittsburg sleep quality index, sleep, sleep questionnaire, Wilson’s disease

## Abstract

**Objective:**

We proposed to detect sleep abnormalities in Wilson’s disease, (WD) using sleep questionnaires.

**Materials and Methods:**

Twenty-five patients (M:F = 18:7; age: 24.4 ± 9.2 years) with WD and 24 controls (all males; age: 33.1 ± 9.7 years) were recruited. They underwent phenotypic/magnetic resonance imaging (MRI) evaluation followed by administration of Pittsburg Sleep Quality Index (PSQI) and Epworth Sleepiness Scale (ESS) questionnaires.

**Results:**

The mean age at presentation and diagnosis was 24.4 ± 9.2 and 17.6 ± 7.5 years, respectively. The duration of illness at diagnosis was 14 ± 21.9 months. On PSQI, 15 patients with WD had abnormal PSQI scores of >5 as compared to 6 patients among the controls. The mean PSQI score was significantly more (*P* = 0.03) in patients compared to the controls. The PSQI worst scores were noted only in WD. Evaluation with ESS showed that three patients with WD scored >10/24, while two among the controls qualified for excessive daytime sleepiness. Overall, assessment by sleep questionnaires detected abnormality in 16 patients with WD as compared to 8 controls (*P* = 0.004). Subgroup analysis revealed that patients whose duration of illness was >8 years and who were on decoppering treatment had significantly lesser excessive daytime somnolence.

**Conclusions:**

Sleep disturbances were observed more often in WD than in controls. Better designed studies will provide a better understanding.

## Introduction

Sleep related disorders are recognized in a third of the Western population. Sleep related disorders affect the mood, behavior, work and quality of life and pose several health problems and contribute to morbidity and mortality of the existing medical illness. Their recognition and treatment can help in improving the functional ability of the individual while preventing hypertension, psychological disturbances and accidents. Sleep disorders could occur in almost all the diseases affecting central nervous system, e.g. extrapyramidal disorders, especially, Parkinsonism.[1] These and other studies support the notion that dopamine activity is normally influenced by circadian factors.[2]

Wilson’s disease (WD) affects young patients, manifests with wide spectra of neuropsychiatric and systemic manifestations, requiring lifelong decoppering therapy and often other symptomatic medications that could affect sleep.[3] The imaging and pathological features of WD suggest that there is affection of the critical areas that are involved in sleep regulation.[4] There are a few studies referring to the sleep disorders in WD. Portala *et al*. (2002), in a study of 26 patients with WD, reported reduced night sleep as one of the 12 most common psychopathologic symptoms.[5] Firneisz *et al*. (2000) reported hypersomnia and sleep onset REM as the main symptom of WD in a 21-year-old man.[6] In view of paucity of such studies in patients with WD, and moreover, WD could be a model among the extrapyramidal disorders, this study was planned.

The aim was to evaluate the frequency and nature of sleep abnormalities in patients with WD, using two validated questionnaires – Pittsburgh Sleep Quality Index (PSQI), Epworth Sleepiness Scale (ESS) in comparison with controls.

## Materials and Methods

The cross-sectional study was carried out from January 2007 to January 2009 on patients diagnosed to have WD in the Department of Neurology at the National Institute of Mental Health and Neurosciences, Bangalore. Twenty-five patients (M:F = 18:7; age at evaluation: 24.4 ± 9.25 years) with confirmed diagnosis of WD were prospectively recruited from the weekly Wilson’s disease clinic and in-patient services of Department of Neurology. Patients known to be suffering from any other medical or surgical condition that can cause sleep disorder and/or on medications (except anti-epileptic drugs) were excluded.

### Study design

Participants were explained the nature of study and an informed consent was obtained from all of them. Ethical clearance was obtained from the institute ethics committee. The study consisted of a) part 1: phenotypic assessment and b) part 2: evaluation with screening sleep questionnaires, namely, PSQI and ESS.

### Control population

Twenty-five healthy controls (all males, age: 33.1 ± 9.7 years) were recruited for the study. They were either the staff of the institute who were not performing shift duties or healthy relatives of in-patients admitted for acquired neurological disorders. Relatives of patients with WD were excluded.

### Drug history

Patients were categorized as drug naïve or on decoppering agents and details of other medications given for the symptomatic treatment of tremor, chorea, dystonia, etc. were also obtained. A patient was considered as drug naïve if he had never received decoppering agents (pencillamine or zinc sulfate).

### Study proper

After recruitment into the study, the subjects were administered the standardized PSQI and ESS.[7,8] One patient could not answer the questionnaire due to severe anarthria and hence was not included for analysis. For ESS, a score of more than10/24 is considered to denote excessive daytime sleepiness. Since all eight questions were not applicable to all the subjects, the percentage of 10/24 (41.5%) was calculated, and if the total ESS score ratio was more than this, it was considered significant. For PSQI, the seven items are scored from 0 to 3 and a composite score out of 21 is calculated. A cut-off score of 5 was treated as abnormal.

## Results

### Demography and phenotype

Twenty-five patients of WD with mean age at evaluation of 24.4 ± 9.25 years (range: 14–62 years) were studied. The duration of illness at diagnosis was 14 ± 21.98 months. The mean duration of illness at evaluation, computed from initial neurological symptom suggestive of WD to the time of recruitment into the study, was 121.33 ± 129.33 months (range: 2–312 months). History of consanguineous parentage was noted in 13/25 patients (52%) and a family history suggestive of WD was noted in 10/25 (40%). Two of these were sibs of patients who had died earlier of hepatic disease, strengthening the diagnosis in index cases. Ten patients (40%) had history of jaundice, suggestive of acute hepatitis in the past. Hepatomegaly and splenomegaly were noted in four and five patients, respectively. One patient had undergone splenectomy due to refractory hypersplenism. Two each had joint pain and abdominal swelling. There was no abnormal elevation of liver enzymes or bilirubin in any of the patients at the time of study. Anemia was present in eight patients. The common neurologic abnormalities were the following: tremor 22 (88%), dysarthria 20 (80%), writing difficulty 16 (64%), Parkinsonism 11 (44%), dystonia 12 (48%), ataxia 8 (32%), pyramidal signs 7 (28%), chorea 3 (12%), seizures 6 (24%), and myoclonus 1 (4%), among others. Six patients (24%) had psychiatric manifestations as their dominant symptoms, which included affective disorders – mania (*n* = 5) and schizophreniform psychosis (*n* = 1). In addition, mental subnormality was observed in 3 (12%) patients.

### Evaluation with sleep questionnaires

#### Epworth sleepiness scale

Three patients and two controls qualified for excessive daytime sleepiness, and the mean ESS score was not significantly different between the groups (patients: 17.5 ± 15.6; controls: 19.3 ± 13.9; *P* = 0.66). Subgroup analysis of the patients revealed that individuals with longer duration of illness (abnormal ESS: <8 years, 3; >8 years, 0) and on decoppering treatment (abnormal ESS: drug naive, 3; on treatment, 19) had significantly lesser “excessive daytime somnolence”. Other variables like gender, young (< 20 years) vs. old (>20 years) age, severity of illness (Chu stage 1 vs. stage 2), and use of anti-epileptic drugs (AEDs) did not influence the scores [Table 1].

**Table 1:** Comparison of the ESS of patients with Wilson’s disease and control subjects

Epworth sleepiness scale	Would never doze		Slight chance of dozing		Moderate chance of dozing		High chance of dozing	
	WD	C	WD	C	WD	C	WD	C
While sitting and reading*	17	12	4	7	1	1		2
Watching TV*	18	25	1	0	3	0		0
Sitting inactive in a public place	19	25	4	0		0	1	0
As a passenger in a car for an hour without a break	16	16	4	7	2	0	2	2
Lying down to rest in the afternoon when circumstances permit	11	7	1	0	5	5	7	13
In a car, while stopped for a few minutes in the traffic*	15	25	1	0		0		0
Sitting and talking to someone	23	24		1		0	1	0
Sitting quietly without alcohol in the afternoon	9	21	6	1	7	0	2	3

* All the questions in the Epworth Sleepiness Scale could not be applied to all the subjects. Q. no. 1 in two cases and three controls; Q. no. 2 in two cases, and Q. no. 6 in eight cases could not be applied ESS score: Patients 17.5 ± 15.6; controls: 19.3 ± 13.9 (*P* = 0.67), WD, Wilson’s disease, C, control subjects

#### Pittsburgh sleep quality index

The seven items of PSQI were scored from 0 to 3 and a composite score out of possible 21 was derived. Fifteen patients had an abnormal PSQI score of >5 as compared to six control subjects (chi-square: 8.3; *P* = 0.004). Among the PSQI subscores, six patients had decreased sleep duration of <6 hours compared to none among the controls (*P* = 0.01). The mean PSQI score was also significantly more (*P* = 0.03) in patients (5.5 ± 3.5) compared to the controls (3.5 ± 2.6) [Table 2].

**Table 2:** Comparison of the PSQI of patients with Wilson’s disease (WD) and control subjects

Parameters	WD	Controls	*P*
Sleep quality			
Good (0, 1)	18	14	0.14
Bad (2, 3)	6	11	
Sleep latency			
<30 min (0, 1)	17	17	0.54
>30 min (2, 3)	7	8	
Sleep duration			
>6 hours (0, 1)	18	25	0.01
<6 hours (2, 3)	6	0	
Sleep efficiency			
>75 (0, 1)	17	21	0.22
<75 (2, 3)	7	4	
Sleep disturbance			
<10 per night (0, 1)	22	22	0.52
>10 per night (2, 3)	2	3	
Sleep medications			
Never in 1 month (0)	24	25	-
Daytime dysfunction			
Never in 1 month (0)	24	25	-
PSQI score of >5	15	6	0.004
Mean PSQI score	5.4 ± 3.5	3.5 ± 2.6	0.03

WD, Wilson’s disease, PSQI, Pittsburgh sleep quality index

## Discussion

The present study involved patients with WD and healthy controls who were administered ESS and PSQI. These tests were validated against polysomnography. The results revealed 64% (*n* = 16) patients with sleep problems, while they were noted in 32% (*n* = 8) of control subjects. The only other study in literature on sleep abnormalities in patients with WD was by Portala *et al*., who had used a different sleep inventory, with 24 patients and 72 controls (1:3). Their study detected sleep abnormalities in 42% of patients.[5] Comparison of the sleep abnormalities on a qualitative basis was not possible due to the disparity in questionnaires used. The reason of high prevalence of sleep abnormality in controls is difficult to explain.

WD is an extrapyramidal disorder with both hyperkinetic and hypokinetic manifestations. The present study found that sleep abnormalities varied as much as the clinical features and shared the features with sleep abnormalities in various other extrapyramidal disorders published in literature.[9–11]

The exact reason for sleep disturbances in WD remains to be ascertained. The distribution of neuronal damage by copper deposition and/or encephalopathy due to hepatic dysfunction is highly variable, and may include brainstem nuclei, dentate nucleus, pons, thalamus, basal ganglia, external capsule, claustrum and frontal lobes as well as a certain degree of diffuse brain atrophy.[12,13] The imaging and pathological features of WD suggest that there is affection of the critical areas involved in the regulation of sleep.[4,14] Copper toxicity could be responsible but its role was not substantiated in this study. REM sleep is generated in the brain stem by acetylcholinergic neurons belonging to the cholinergic system (“REM-on” cells). Activation of noradrenergic neurons of locus coeruleus and serotoninergic neurons of dorsal raphe nuclei belonging to the monoaminergic system (“REM-off” cells) inhibits the cholinergic system. Patients with WD may have impairment in the monoaminergic system. An abnormal metabolism of neurotransmitters, probably due to an increase in the activity of copper containing enzymes like dopamine ß-hydroxylase,[15] with increased noradrenaline in the striatum, has been reported.[16,17] Moreover, Nijeholt (1978) reported a decrease both of 5-hydroxyindole acetic acid (5-HIAA, a 5-HT metabolite) and homovanillic acid (HVA, a metabolite of dopamine) in the lumbar CSF of patients of WD, before and during treatment with penicillamine.[18]

The sample size was small and consisted of a heterogeneous group. A study involving larger number of subjects with more qualitative description of various sleep abnormalities may throw light into the nature of sleep abnormalities in these patients.
